# The Role of Parenting Interventions in Optimizing School Readiness for Children With Disabilities in Low and Middle Income Settings

**DOI:** 10.3389/fped.2022.927678

**Published:** 2022-06-28

**Authors:** Tracey Smythe, Nihad A. Almasri, Marisol Moreno Angarita, Brad D. Berman, Olaf Kraus de Camargo, Mijna Hadders-Algra, Paul Lynch, Maureen Samms-Vaughan, Bolajoko O. Olusanya

**Affiliations:** ^1^International Centre for Evidence in Disability, Department of Population Health, Faculty of Epidemiology and Population Health, London School of Hygiene and Tropical Medicine, London, United Kingdom; ^2^Division of Physiotherapy, Department of Health and Rehabilitation Sciences, Stellenbosch University, Cape Town, South Africa; ^3^Department of Physiotherapy, The University of Jordan, Amman, Jordan; ^4^Research Group on Disability, Policies and Social Justice, School of Medicine, Universidad Nacional de Colombia, Bogotá, Colombia; ^5^UCSF Benioff Children's Hospital, San Francisco, CA, United States; ^6^CanChild Centre for Childhood Disability Research, McMaster University, Hamilton, ON, Canada; ^7^University of Groningen, University Medical Center Groningen, Department of Paediatrics, Institute of Developmental Neurology, Groningen, Netherlands; ^8^School of Education, University of Glasgow, Glasgow, United Kingdom; ^9^Department of Child and Adolescent Health, University of the West Indies, Mona Campus, Kingston, Jamaica; ^10^Centre for Healthy Start Initiative, Lagos, Nigeria

**Keywords:** children with disabilities, school readiness, parenting interventions, low- and middle-income countries, inclusive education

## Introduction

Children with disabilities include those who have long-term physical, mental, intellectual or sensory impairments that in interaction with various barriers may hinder their full and effective participation in society on an equal basis with their non-disabled peers (1, 2). The protection of children with disabilities is enshrined in the UN Convention on the Rights of the Child (3), Convention on the Rights of Persons with Disabilities (UNCRPD) (2), and the Sustainable Development Goals (SDGs) (4). There is a global commitment to move toward Universal Health Coverage (UHC) as part of efforts to achieve Sustainable Development Goal 3 to “ensure healthy lives and promote wellbeing for all at all ages”. The actions of nations to realize the rights of health for all will also impact the probability of achieving SDG target 4.2, which requires, “all girls and boys have access to quality early childhood development, care, and pre-primary education so that they are ready for primary education”. The right to education applies to all children, and Article 24 of the UNCRPD (2) ensures that children with disabilities attend their local school and that schools accommodate their specific needs. In signing these pledges, governments have committed to addressing inequity for children with disabilities through the provision of disability-inclusive education and appropriate health services.

Fulfillment of these pledges is important, especially for the 53 million children <5 years of age with developmental disabilities, which include, but are not limited to, epilepsy, intellectual disability, sensory impairments, autism spectrum disorder or attention deficit hyperactivity disorder (5). They face greater challenges accessing quality healthcare services and experience worse health outcomes, especially in low- and middle-income countries (LMICs) (6). Furthermore, a focus on supporting children with disabilities to thrive and transform during their early years is important as this period is critical for maximizing their personal development and achieving their learning outcomes and readiness for school (7). Fulfillment of these pledges requires financial and strategic investment: It is equally essential that the global community recognizes practices of exclusion. For example, in LMIC the likelihood of a child having an impairment before their fifth birthday was 10 times higher than the likelihood of dying in 2019 (8) and yet integration of inclusive health and education services for children with disabilities remains inequitably deficient (9, 10).

For the purpose of this paper, we use the term “early child development” (ECD) to include health, physical, social, emotional, cognitive and language development in the first 5 years of life (11). Within interventions to promote ECD, we use the term “parenting interventions” to encompass social and behavioral techniques or training that include any primary caregiver of a child with a disability. We refer to school readiness as a child's adequate preparation to engage in a school environment and activities, interconnected with school practices that foster a smooth transition to primary school and parental attitudes toward the school and support for early learning (12). In the context of school readiness, we define parenting interventions as skills training to assist parents in better supporting children with disabilities at home and preparing them for school. We use the term “inclusive education” to mean that different and diverse learners are welcomed and taught side by side with their peers and enjoy safety and participation with informed parental decision-making. It should be delivered in supportive environments in which “all members of the community are welcomed equally, with respect to (the different types of) diversity” (2).

Children with disabilities have limited access to ECD services in resource limited settings; moreover current ECD services are not designed to meet their specific learning, physical, and communication needs (13). It follows that ECD services experience challenges in enabling school readiness for children with disabilities. In this article we propose that culturally sensitive parenting interventions should enable and better inform parents, in partnership with ECD training programs at schools, to improve opportunities for children with disabilities to achieve their optimal potential and thrive in school. Ultimately, strengthening global early child initiatives and health and education systems toward achieving human rights and global development goals for all.

## The Importance of the Early Years

Responsive parent-child relationships, and combined parental and community support for learning during the earliest years of life are recognized as being crucial for promoting successful ECD (14). By attending to the child's and the family's needs and strengths, the provision of appropriate and culturally relevant support and services can improve child and family outcomes. This requires the community, teachers and parents to work together with the child toward the successful and inclusive acquisition of individual and desired developmental and learning outcomes (12). These foundations for learning are largely built in the early years of life before a child participates in primary school education. Children who fall behind during these early years seldom catch up with their peers, perpetuating a cycle of underachievement and high dropout rates that continues to harm marginalized children and adolescents (15).

## Current State of Inclusive Education and Child Health

**Figure 1:**
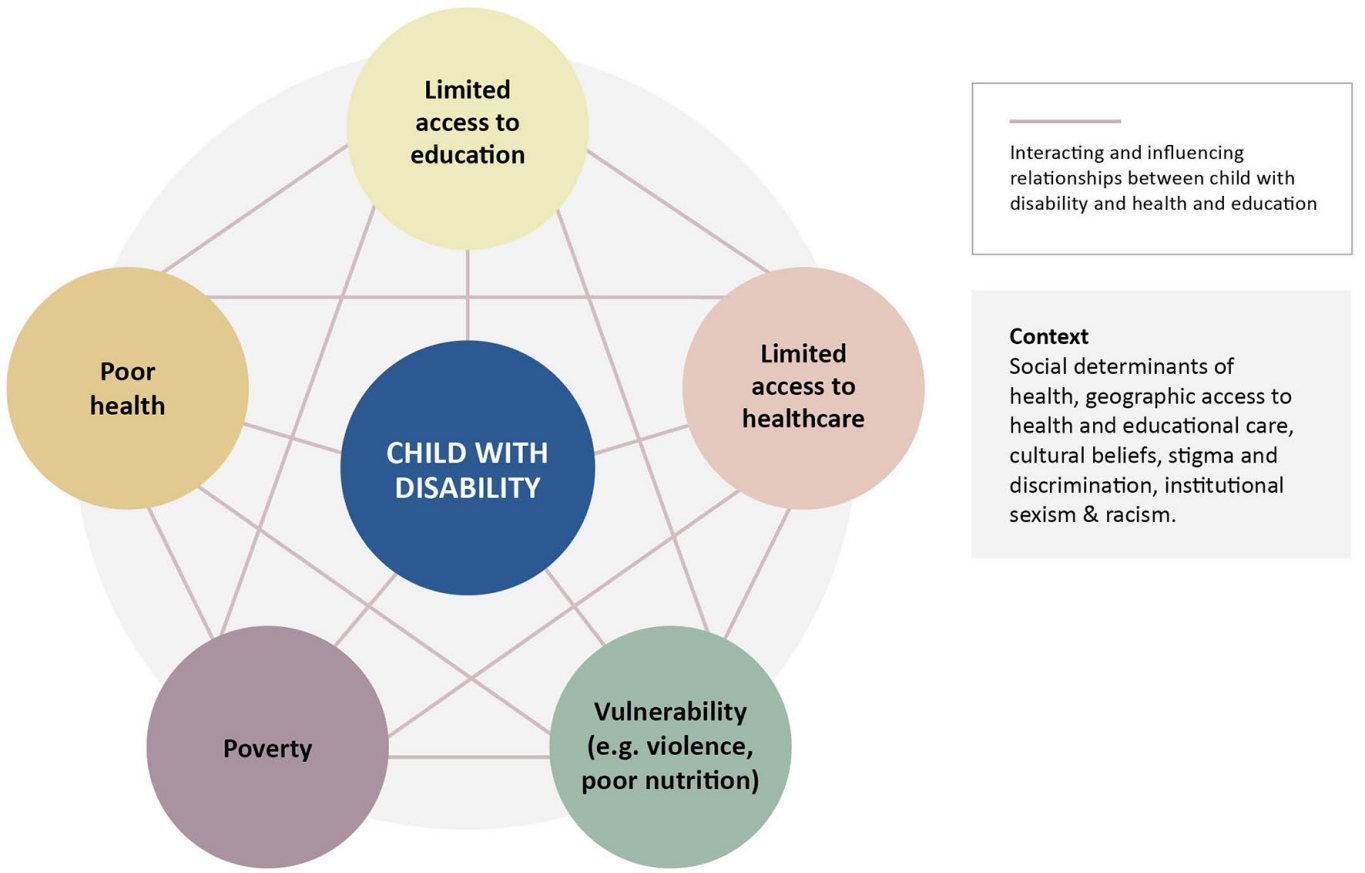
Interrelationship between disability, poor health and limited access to education.

The interrelationship between disability, health and early childhood education is complex. These constructs are overlapping, intertwined and reinforcing, and place children with disabilities in vulnerable situations. In Figure 1, we propose a conceptual model based on the current evidence that demonstrates the reinforcing cycle of disability and poverty (16), which in turn limits access to education and health care (1, 17).

Limited access to quality education can have adverse effects on health (e.g., lack of access to school lunch), expose children to violence (e.g., increase of domestic violence during COVID pandemic) (18) and can result in greater adversity. Contextual factors create multiple barriers to successful inclusion as a result of social determinants of health, geographic location, cultural beliefs, discrimination and institutional sexism and racism (19). Thus, a child with disability is vulnerable to poor nutrition (20) and violence (21), at risk of poverty (16) and poor health and wellbeing (22), and has limited access to health care (17) and education (23). These factors are not only linked with disability, but are also interrelated as shown in the diagram.

The relationship between disability, education, health and social protection will not be the same for all; children with disabilities are a highly diverse and heterogeneous group. Nevertheless, they experience limited access to education, including ECD centers, as these centers are not necessarily able to accommodate the needs of children with disabilities through their curriculum programs (24). Disparities also exist between regions of the world in how supports are established for children with disabilities and their families. Health-care systems in high-income countries often support the early diagnosis and intervention with such children, including parenting interventions (25). In LMICs where the majority of children with disabilities live (5), there remains a lack of availability for continuous care and a dearth of information about ECD interventions, including parenting interventions. This paucity of knowledge is perpetuated through research agendas. For example, a recent global systematic review (26) excluded parenting interventions for children with disabilities, and a call for action found that 50% of the registered clinical trials reviewed explicitly excluded young children with disabilities (27, 28). Research agendas are also influenced by global funding for grand challenges and policy makers and there remains a gap in government funding and development assistance for children with disabilities and their families in LMIC, relative to population needs and epidemiology (29, 30).

Optimal support for early child development has lifetime beneficial consequences for educational achievement, adult productivity and population health (27). Despite the wellrecognized importance of investment in children's formative years, only half of the world's preschool-aged children attend pre-primary programs (from age 3 years up to the start of primary education, often aged 6 years) (31). Whilst formal education usually starts between 5 and 6 years in many LMICs, only 2 in 10 children have the privilege of attending pre-primary programs and barriers are even higher for marginalized groups such as children with disabilities (31). These barriers include a limited accommodation process, physical inaccessibility, negative attitudes, stigma and discrimination, local cultural beliefs, expectations about diverse functionalities and curricula that do not address the needs of children with disabilities (32, 33). It is timely that UNICEF's draft strategic plan (2022–2025) includes a focus on supporting caregivers, communities and schools to provide the environments, care, protection and education that enable children's health, nutrition and development (34). The smooth transition for children to primary education often referred to as “school readiness”, requires the school, families/communities and children to work together well before enrolment into school to facilitate communication abilities along with peer social behaviors. The young child's readiness for school focuses on learning and developmental outcomes; the school's readiness for the child usually focuses on school-level outcomes and practices that foster a smooth transition into primary school; and families' readiness for school usually incorporates parent attitudes to the school itself and involvement in the child's early learning as well as development and transition to school (35). Ready schools, parents, children and communities need to work together to achieve this smooth transition. Still, the link between ECD initiatives and school readiness remains contentious as there is a tendency to focus on pre-academic skills (early literacy and numeracy), even at home. Children with disabilities may not be able to achieve pre-academic skills in time or at all, but are more focused on daily living activities (e.g., be toilet trained and able to feed themselves). There remain inequities in the delivery of services for some children with disabilities, and initiatives approach groups of children differently, depending on the functionality and severity of their impairment. (E.g., a child with multiple physical and cognitive disabilities may not be able to participate in many learning and play activities without adequate support.).

Children with disabilities in LMICs can be excluded from school due to parent and caregiver fear of not being able to provide adequate care, teachers being overwhelmed with the presence of children with disabilities and lack of training on inclusion, or classes being simply too large to pay proper attention to the child's needs (24). Families of children with disabilities are often unable to visit the school before the start of the academic year, thus creating significant difficulties for a smooth transition into school for the child. Children with disabilities are further marginalized by the design and structure of school curricula, and by perceptions of their limited abilities (32). Finding ways to meet the individual learning, social, and physical needs of students with disabilities can be challenging in schools and contexts with severely limited resources (32). For example, a child with cerebral palsy may require a modified chair with a flat top with an edge on which to place and move objects. There should be opportunities for ready schools, parents, children and communities to develop the necessary relationships and information sharing that will support a smoother transition for children with disabilities, from the early child-center into primary school, where all are then ready to engage together (12).

## The Role of Parenting Interventions in Optimizing School Readiness

There are broadly two approaches to providing ECD for children with disabilities, including them in mainstream ECD interventions, and targeting interventions according to their individual needs. There remains a need for inclusive approaches for children with disabilities in mainstream services, as well as within specialist ECD interventions. This means that the role of parents can be particularly crucial to fill existing gaps in service availability. A key factor in optimizing a child's school readiness, is fostering the capabilities of parents and communities to help to scaffold development and early learning. Many interventions that are designed to boost school readiness target parents and parenting skills. Interventions include approaches that increase parent wellbeing, knowledge and confidence, as well as enhance parent sensitivity and responsiveness, enrich parent-child communication and increase parent support for early learning through skills training programs. For example, parenting interventions for children during the first 3 years of life lead to improvements in early cognitive, language, motor, socioemotional development, and reduce child behavior problems across LMICs and high-income countries (26). Parenting interventions also improve parenting knowledge and practices, and parent–child interactions (26, 36, 37).

Accordingly, parenting interventions that include skills training to assist parents in being better able to care for a child with a disability at home and prepare them for school may contribute to optimizing readiness for school. Children with disabilities would benefit from these parenting interventions to improve their child's chances of being school ready and address the challenges that they may face, which are often compounded by community attitudes and beliefs espoused in relation to disability (33). Notably, combining the education of parents and the training of teachers has a greater impact on child outcomes, such as increased language skills (38), and may also benefit social development, improved play and motor skills (39). There is, therefore, a need to deliver parallel training at the school level on the development of pedagogy, teacher skills and positive attitudes to inclusion. Nevertheless, the current policy landscape for ECD has not yet resulted in greater investments and implementation of large-scale national parenting programs and critiques of parenting interventions in LMICs raise ethical challenges and concerns (40, 41).

## Critique of Parenting Interventions in Low Resourced Settings

Parenting intervention practices are commonly derived from attachment theory and responsive care, in which the quality of attachment stems from the way that a mother cares for her child, and are presented as the universal standard of good care (40). These practices are typically Euro-American constructs and include little attention to community practices. Such parenting interventions involve encouraging caregivers to change their practices and views, usually with little understanding of how such changes affect child, family, and community (40). Typically, in contexts of limited support and scarce resources, there may be a combined collective input of all caregivers, rather than only the mother or grandmother who assume responsibility for the child's development and welfare. We need to carefully consider existing beliefs, practices, stigma and developmental goals in the targeted communities to ensure that ethical principles are fulfilled (41) all the while preserving the efficacy of parental support for their children within the communities they live in.

There is a clear narrative for inclusive child health, education, and protection (safeguarding) in LMICs; children with disabilities are excluded from recommendations, initiatives and policies. The lack of investment in inclusive education is reflected in low service level inputs and consequently education coverage and outcomes for children with disabilities. Investment in education and scale-up of services for children with disabilities is needed urgently. For instance, the percentage of Gross Domestic Product spent on education in 2019 is 5.3 in North America, but only 3.5 in sub Saharan Africa and 2.5 in South Asia (42). UNESCO's recommendation for government budget for education by 2030 is 4–6% of GDP and/or the allocation of at least 15–20% of public expenditure (43). It is estimated that an additional 50 cents per person annually is the cost for ECD to be incorporated into existing services (44). This level of funding for education, if followed through in LMIC, will translate to significant improvement in public investment in education with measurable outcomes. Partnership between policymakers and educators should support governmental investment in ECD and implementing effective and culturally appropriate parenting interventions.

## Multidimensional Roadmap for Inclusive Education: Priorities for Parenting Interventions and School Readiness

Children with disabilities should be able and have a right, to experience positive wellbeing and full involvement at school rather than merely attending education services. Parents of children with disabilities experience emotional distress, isolation and lack of support, particularly in cultures where unfavorable superstitious beliefs about disability prevail (45), which may trigger profound disappointment, prolonged grief and a sense of hopelessness for a seemingly uncertain future for their children (46). Parenting interventions, for school readiness designed for parents to be able to better care for a child with a disability at home and prepare them for school, may not only instill hope of a better independent and productive living but are also reassuring to the entire family. The role of culturally sensitive parenting interventions in tandem with ready schools and inclusive communities that are supported by the policy is critical for achieving inclusive and quality education. The intervention needs to be family-centered and ensure that families are more confident to discharge their role effectively. Parenting interventions for parents of children with disabilities should recognize the inherent value of the experience that the parent possesses. They need to be respectful of how children are brought up and educated in the local culture, and build on the local practices, knowledge and strengths that exist in early child education whilst collaborating with local training providers, community and ECD services (24). Active engagement between the health, social welfare and education sectors at all levels is required. Within schools, additional support needs to be provided to support children with disabilities, through assistance to help with toileting, feeding, mobility, communication and safe play. We need to scaffold child development and learning at their level and enrich current parent intervention approaches to work together with training of teachers. This may go some way to preventing the growing gap in provision of early learning and contribute toward achieving the full intent of SDG 4.2.

## Call to Action

As ECD specialists, health professionals and researchers committed to equity and social justice, our task is to reveal patterns of avoidable differences experienced by children with disabilities in accessing inclusive and equitable quality education. We strongly recommend the implementation of system-wide strategies to address the prevailing inequities and barriers, such as the lack of education resources for parents, the lack of training and appropriate resources for preschool professional staff and more importantly the implementation of inclusive education policies by the education sector, which continue to shift the onus to children with disabilities and their families. Close examination of the readiness and capacity of a nation's schools to receive all young children and support their learning and development is needed. Inclusion demands that educators and policymakers consider two key questions when reviewing policies and practices: (1) Who benefits? (47) and (2) Inclusion into what? (48). We call for accelerated political will and action to adapt and deliver parenting interventions for children with disabilities, which are respectful of diverse local contexts, whilst coordinating within existing systems and services. The current evidence suggests that parenting interventions are effective for ECD however, most studies are conducted in high-income settings, which raises questions about generalizability; complementary investment in addressing the needs of the beneficiaries of child survival programs with lifelong impairments in LMICs is required.

## Conclusion

Children with disabilities face sustained inequities despite the international agenda that supports inclusion. This is perpetuated through exclusive early child initiatives and policies, practice, and research. The global agenda urgently needs to move beyond token recognition of this marginalized group to inclusive early child intervention programs that consider existing practices, cultural beliefs, and developmental goals in the targeted communities. Children with disabilities in LMICs should receive culturally sensitive parenting interventions to improve learning and educational outcomes. These initiatives must be geared toward “school readiness” for educational inclusion of children with disabilities and this necessitates that the community, teachers, and parents work together with children toward successful developmental and learning outcomes. Culturally sensitive parenting interventions, and early child development teaching programs at schools, may thus contribute to strengthening education systems toward achieving the full intent of SDG 4.2 and human rights global development goals for all.

## Author Contributions

All authors listed have made a substantial, direct, and intellectual contribution to the work and approved it for publication.

## Conflict of Interest

The authors declare that the research was conducted in the absence of any commercial or financial relationships that could be construed as a potential conflict of interest.

## Publisher's Note

All claims expressed in this article are solely those of the authors and do not necessarily represent those of their affiliated organizations, or those of the publisher, the editors and the reviewers. Any product that may be evaluated in this article, or claim that may be made by its manufacturer, is not guaranteed or endorsed by the publisher.
